# Differential interactome mapping of aggregation prone/prion-like proteins under stress: novel links to stress granule biology

**DOI:** 10.1186/s13578-023-01164-7

**Published:** 2023-12-01

**Authors:** Neelam Younas, Saima Zafar, Tayyaba Saleem, Leticia Camila Fernandez Flores, Abrar Younas, Matthias Schmitz, Inga Zerr

**Affiliations:** 1 Department of Neurology, University Medical Center, Georg-August-Universität, Robert-Koch-Strasse 40, 37075 Göttingen, Germany; 2 German Center for Neurodegenerative Diseases (DZNE), Robert-Koch-Straße 40, 37075 Göttingen, Germany; 3 Biomedical Engineering and Sciences Department, School of Mechanical and Manufacturing Engineering (SMME), National University of Sciences and Technology (NUST), Islamabad, Pakistan

**Keywords:** Stress granules, Oxidative stress, Neurodegenerative diseases, Interactomics, Prion/prion-like proteins

## Abstract

**Background:**

Aberrant stress granules (SGs) are emerging as prime suspects in the nucleation of toxic protein aggregates. Understanding the molecular networks linked with aggregation-prone proteins (prion protein, synuclein, and tau) under stressful environments is crucial to understand pathophysiological cascades associated with these proteins.

**Methods:**

We characterized and validated oxidative stress-induced molecular network changes of endogenous aggregation-prone proteins (prion protein, synuclein, and tau) by employing immunoprecipitation coupled with mass spectrometry analysis under basal and oxidative stress conditions. We used two different cell models (SH-SY5Y: human neuroblastoma and HeLa cell line) to induce oxidative stress using a well-known inducer (sodium arsenite) of oxidative stress.

**Results:**

Overall, we identified 597 proteins as potential interaction partners. Our comparative interactome mapping provides comprehensive network reorganizations of three aggregation-prone hallmark proteins, establish novel interacting partners and their dysregulation, and validates that prion protein and synuclein localize in cytoplasmic SGs. Localization of prion protein and synuclein in TIA1-positive SGs provides an important link between SG pathobiology and aggregation-prone proteins. In addition, dysregulation (downregulation) of prion protein and exportin-5 protein, and translocation of exportin-5 into the nucleus under oxidative stress shed light on nucleocytoplasmic transport defects during the stress response.

**Conclusions:**

The current study contributes to our understanding of stress-mediated network rearrangements and posttranslational modifications of prion/prion-like proteins. Localization of prion protein and synuclein in the cytoplasmic SGs provides an important link between stress granule pathobiology and aggregation-prone proteins. In addition, our findings demonstrate nucleocytoplasmic transport defects after oxidative stress via dysregulation and nuclear accumulation of exportin-5.

**Supplementary Information:**

The online version contains supplementary material available at 10.1186/s13578-023-01164-7.

## Background

Neurodegenerative diseases are a growing public health problem worldwide. The current lack of effective treatments challenges our understanding of these neurodegenerative maladies.

Although the pathology-related proteins in multiple neurodegenerative diseases are different e.g. prion protein (PrP) in prion diseases, tau and Aβ in Alzheimer’s disease (AD), and synuclein in Parkinson’s disease (PD); one common feature of these maladies is the accumulation of oligomeric and amyloidogenic protein aggregates [1, 2]. Aberrant interactions between proteins result in abnormal deposition of self-aggregating misfolded proteins with the formation of higher-ordered insoluble fibrils in different neurodegenerative disorders. The exact mechanisms of abnormal folding remain enigmatic; however, mounting evidence leads to the presumption that a combination of genetic and environmental factors (particularly oxidative stress) are involved [3, 4]. Emerging evidence supports the hypothesis that oxidative stress combined with protein aggregation initiates a cascade of events, eventually leading to cell death in many neurodegenerative disorders [4].

Recently, pathological and persistent stress granules have been linked to a subset of neurodegenerative disorders including Amyotrophic Lateral Sclerosis (ALS) and AD [5, 6]. ALS provides the most convincing link between SGs and neurodegeneration, as many of the proteins present in the pathological inclusions are also present in transient stress granules, induced in normal cells in response to stressful conditions to promote cell survival. The recent discovery of stress-mediated tau hyper-phosphorylation and oligomerization as a part of the endogenous translational stress response has revolutionized the field of protein aggregation disorders [7]. Aberrant and pathological/persistent stress granules have become prime suspects in the nucleation of toxic protein aggregation [8]. In the current study, we used two cellular models (SH-SY5Y and HeLa cells) to induce oxidative stress using sodium arsenite (NaAsO_2_), a well known inducer of oxidative stress and stress granules [9]. Several models have been used to understand stress granules at cellular level [10]. The human SH-SY5Y neuroblastoma cell line is a classic in vitro model to study AD [11] and PD [12, 13]. Additionally, HeLa Cell model is an alternative cell line and previous investigations have proved their utility in the study of neurodegenerative diseases such as AD and PD[14–17]. A significant amount of knowledge on SGs originates from investigations in HeLa cells, including 154 studies published between 1999–2014 [10]. Although, this is a non-neuronal cell line, this model worked for this exploratory study as big cytoplasmic area of HeLa cells in comparison with other cell lines permits accurate identification of cytoplasmic stress granules [18].

Gaining more insights into the biological processes and pathways associated with neurodegenerative disease-related proteins, and their reorganization under oxidative stress is crucial for understanding pathophysiological molecular events linked with these proteins. The exploration of various stress conditions, as well as parallel datasets showing the differences and similarities between protein–protein interactions and functional networks are missing [19]. A powerful approach to increase our knowledge about these cellular processes is the use of high throughput proteomic technologies, which not only allow analyses at the protein level but also at post‑translational levels, which cannot be discovered by gene-based approaches. Mass spectrometry (MS) analysis is a well‑suited methodology to unveil complex molecular networks. Among them, label‑free MS approaches are simpler (in terms of sample preparation) and lower in cost [20, 21]. The combination of immunoprecipitation with MS technologies allows rapid and specific identification of distinct partners of the protein–protein interaction networks. Notably, this IP-MS approach has identified interactors that had previously been shown to interact with the bait of interest using other technologies, reinforcing the efficacy of the methodology [22]. Taken together, co-immunoprecipitation-MS (CO-IP/MS) approach is a reliable, rapid and sensitive method to identify protein interaction partners, and is very useful for the identification of novel interacting protein members that cannot be discovered using conventional approaches [22].

To this end, in the current study, we used a combination of co-immunoprecipitation and mass spectrometry (MS) to compare the interactome rearrangements of neurodegenerative disease-linked proteins (PrP, synuclein, and tau) under basal and oxidative stress-induced conditions; and the impact of these findings on the understanding of pathophysiological processes.

## Results

### Prion protein and synuclein localize in TIA1-positive stress granules

Aberrant stress granules have been linked with the aggregation of many neurodegenerative disease-associated proteins including tau protein [7, 23]. Stress granules are formed via a process called liquid–liquid phase separation (LLPS). Prion protein has most of the physicochemical properties to carry out LLPS, including a significant level of intrinsic disorder and the ability to interact with RNA [24].

**Fig. 1:**
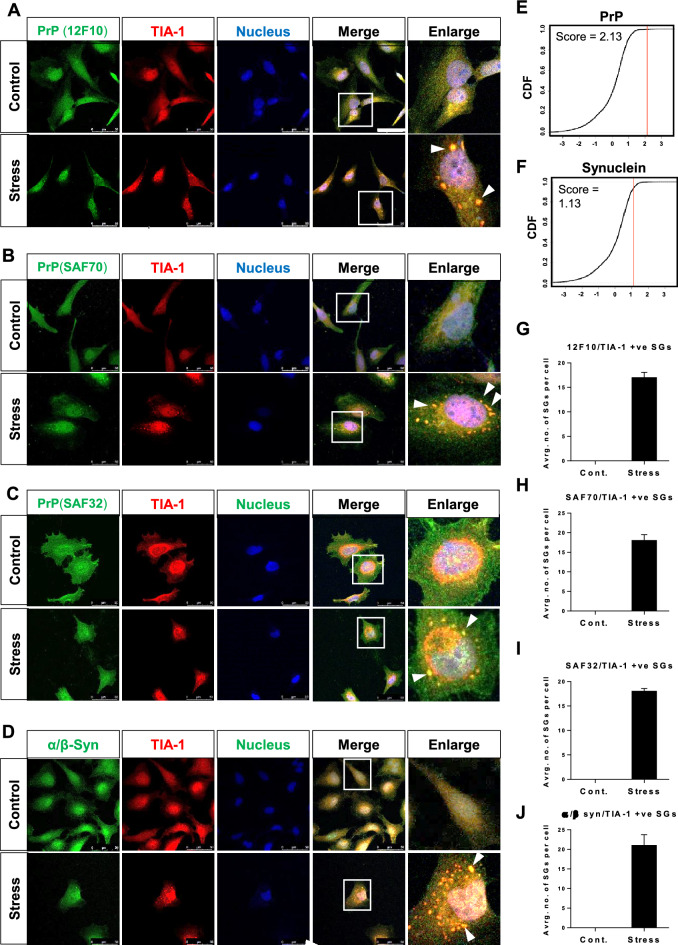
Localization of PrP and α/ꞵ synuclein in TIA1-positive stress granules after oxidative stress induction in HeLa cells. **A-D** Representative immunofluorescence results**.** Localization of endogenous TIA1, PrP, and α/β synuclein was investigated in sodium-arsenite-treated {(0.6 mM; 60 min) (stress)} and untreated (control) HeLa cells using co-immunofluorescence and confocal microscopy. Counter-staining was performed to visualize cell nuclei, scale bar = 50 μm. White arrows show yellow cytoplasmic dots with TIA1 and PrP co-localization in granules. Three different antibodies were used against prion protein (SAF 32, SAF 70, and 12F10). **E** and **F** The granule formation propensity of PrP, and synuclein was assessed by the catGRANULE algorithm. **G**-**J** Quantification of the number of TIA1-positive stress granules

So, firstly we sought to explore the granule formation propensity of PrP and synuclein, using the catGRANULE algorithm [25]. A score of 2.13 was identified for PrP and 1.13 for synuclein protein (Fig. 1E & F). To ascertain whether endogenous PrP and synuclein protein can actually form granules, we co-stained them with a well-known stress granule marker, TIA1 (Cytotoxic granule associated RNA binding protein TIA1) in stress-induced and control (untreated) cells. In the current study, sodium aresnite, a well know inducer of oxidative stress was used for stress induction [26].

The PrP was localized both in the cytoplasmic and nuclear compartments in the control HeLa cells (Fig. 1A), in agreement with previous findings [27–30]. Although, it is well know that PrP is a cell surface protein, it has been also localized in the nucleus of neuronal and endocrine cells [27–30]. PrP also interacts with several intracellular proteins, most of them are found in the cytosol, mitochondria, and nucleus [30, 31].

Stress-treatment (oxidative stress, 0.6 mM sodium aresnite, 60 min) induced cytoplasmic foci of PrP (Fig. 1A). To find out whether or not the PrP granules were actually stress granules, we co-stained HeLa cells with TIA1. There was partial colocalization between PrP and TIA1, in the control cells. After oxidative stress treatment, co-localization was particularly observed between PrP and TIA1 in the form of granules (yellow foci in the cytoplasm) (Fig. 1A, enlarged view). The amount of cytoplasmic TIA1 signal was low because labeling only detected endogenous TIA1, and TIA1 that was present in the cytoplasm was largely in the form of inclusions. Three different antibodies were used against prion protein (SAF32, SAF70, 12F10) to confirm the localization of PrP in stress granules (Fig. 1A-C, G-I).

The synuclein protein was relatively highly enriched in the nucleus with a punctate pattern in the cytoplasm in control cells, in agreement with previous findings in Hela and SH-SY5Y cells [17, 32–35]. Alpha-synuclein can be found in the nucleus [36] and it’s nucleus localization is regulated by numerous factors, including post-translational modifications [34, 37] and oxidative stress [32, 33, 38]. The synuclein protein exhibited a change in its sub-cellular localization upon sodium aresnite treatment (stress), forming cytoplasmic foci that overlapped with stress granules (Fig. 1D and J).

Overall, these findings indicate that stress induces cytoplasmic PrP and synuclein inclusions, which co-localize with TIA1-containing stress granules.

### Comparative interactome mapping identifies distinct and converging molecular pathways

Next, to investigate stress-induced alterations in the interactome of these prion/prion-like proteins, we performed comparative interactomics under control and oxidative stress conditions. Parallel analysis of three prion/prion-like proteins (PrP, synuclein, and tau) using an identical workflow, provided a unique opportunity to compare their interactomes. To find out the physiologically relevant interactor of bait proteins endogenous, native, and untagged proteins were successfully immunoprecipitated (Additional file 1: Fig. S1 A & B). In immunoblotting analysis of tau-immunoprecipitates, cleavage fragments of tau protein were also observed (Additional file 1: Fig. S1B) in agreement with previous studies [39–41]. A significant portion of endogenous tau protein is present in the form of proteolytic fragments (< 45 kDa) in the human brain [39–41]. These bands were not observed in the input as the expression of endogenous tau is quite low, while immunoprecipitation led to enrichment of tau species and the truncated forms of tau were detectable in the immnunoblotting analysis (Additional file 1: Fig. S1B)’’.

Proteins were considered specific interaction partners when they [1] were only identified in the interactome of target proteins and absent in the negative control or [2] showed statistically significant enrichment in comparison with negative controls (Additional file 2). In total, 597 proteins were classified as potential interacting partners (passed our cutoff criteria) (Additional file 1: Fig. S1C & Additional file 2). It should be noted that some of the identified potential interaction partners may be indirect interactors that require other proteins for binding to the bait proteins.

**Fig. 2:**
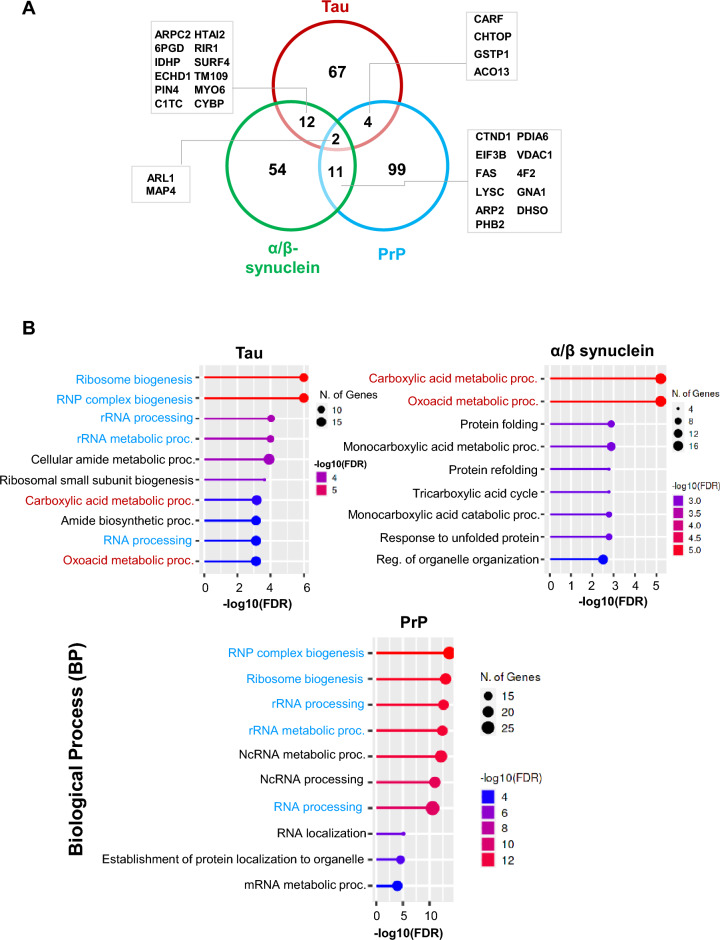
Differential interactome of tau, α/ꞵ synuclein, and PrP protein identified by co-immunoprecipitation combined with mass spectrometry analysis in HeLa cells. **A** A Venn diagram is showing unique and shared proteins in all three bait proteins identified under oxidative stress stimuli. **B** Gene ontology (GO) analysis in the domain of ‘’Biological Process (BP)’’ from ShinyGO webtool, in all three, bait proteins. The significance of the gene ontology enrichment is represented by the negative logarithm of the FDR (FDR < 0.05). The blue highlighted terms are shared between tau and PrP interactome and the red highlighted terms are between tau and synuclein interactome

There were 280 proteins that were specifically identified under stressful conditions (sodium aresnite, 0.6 mM, 60 min), in all three bait proteins (Fig. 2A & Additional file 2). Among these potential interactors, two proteins (ARL1, MAP4) were shared in all three investigated bait proteins (Fig. 2A).

Functional enrichment analyses in the domain of biological process (BP) showed that the protein networks from each individual bait protein organized into several distinct and converging functional categories (Fig. 2B). Based on functional profiles of enriched GO-terms, the major similarity was discovered between interactome make-up of tau and prion protein after oxidative stress induction, in vitro. Five protein categories including ‘‘ribonucleoprotein complex biogenesis’’, ‘‘ribosome biogenesis’’ and ‘‘rRNA processing’’ were specifically enriched in tau- and PrP-interactomes. The protein categories related to metabolism including ‘’carboxylic acid metabolic process, oxoacid metabolic processes’’ were shared between tau and synuclein interactomes (Fig. 2B).

### Stress-induced remodeling of tau interactome to regulate oxidation–reduction and RNA-processing

To understand network rearrangements in tau interactome after stress induction, we performed a comprehensive comparative analysis between control (non-stressed) and stress-induced cells (0.6 mM sodium aresnite). Overall, we identified 143 potential interaction partners of tau protein (Additional file 1: Fig. S3 A & Additional file 2), (i) stress-dependent partners (77 proteins) that associate with tau protein only upon oxidative stress induction (ii) stress-independent interactors (8 proteins), which associate with tau protein independently of stress, and (iii) stress-sensitive interactors (58 proteins), which were lost after stress induction (Additional file 2). The identified proteins include known tau-interacting partners (53 proteins), validating our results. Importantly, a majority of the other identified proteins represent previously uncharacterized tau interacting factors (Additional file 2).

**Fig. 3:**
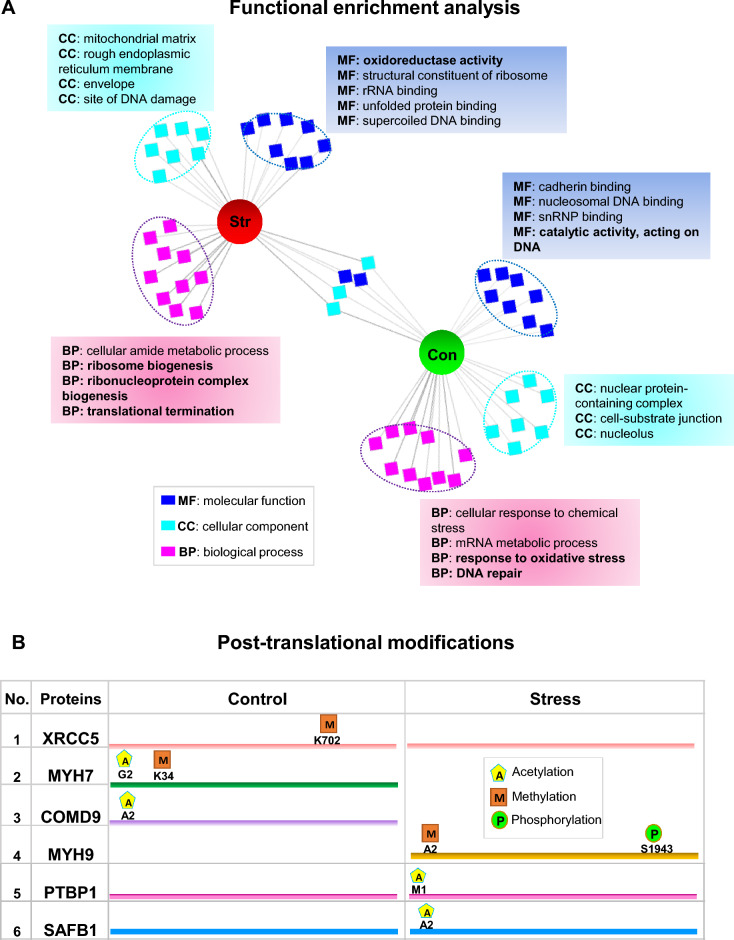
Stress-induced alterations in the tau-interactome **A** Functional enrichment analysis of GO-terms in the domains of biological process (BP), molecular function (MF), and cellular component (CC) was performed using the ToppCluster web tool. An ‘abstracted’ network option was chosen to generate a Cytoscape-compatible network, containing the top ten enriched terms in the tau-interactome from each control (con.) and stress-induced (Str.) condition. Some of the specific terms are labeled in the figure. **B** Post-translation modifications (acetylation, methylation, and phosphorylation) were identified in the tau-interactome of control and stress-induced (stress) cells by MS analysis

To further explore systematically, if a particular molecular function, biological process, or cellular component was enriched in the interactome that preferentially co-isolated with tau protein under stressful conditions; a comprehensive GO search was conducted using ToppCluster (Fig. 3A). The top-ranked categories include ‘‘oxidoreductase activity, ribonucleoprotein complex biogenesis’’, ‘‘translational termination’’ in stress-induced cells (Fig. 3A); consistent with previously characterized functions of tau [7]. Interestingly, one category that was exclusively enriched in control was “DNA repair” indicating an important physiological role of tau in the cell (Fig. 3A).

In the current study, we also investigated three post-translational modifications ꟷphosphorylation, methylation, and acetylationꟷby MS analysis. Among these modifications, phosphorylation, and acetylation were the most frequent modifications. A map of those post-translational modifications, which were exclusively identified in either control or stress-induced (stress) cells is shown (Fig. 3B). Methylation (at residue K702) on X-ray repair cross-complementing protein 5, acetylation (G2) and methylation (K34) on Myosin-7 and acetylation (A2) on COMM domain-containing protein 9 was exclusively identified in control cells. Methylation (A2) and phosphorylation (S1943) on Myosin-9 protein, acetylation (M1) on polypyrimidine tract-binding protein 1 and acetylation (A2) on scaffold attachment factor B1 was exclusively identified in stress cells (Fig. 3B).

### Oxidative stress-induced changes in the synuclein-interactome related to cell-redox and metabolic processes

For the identification of synuclein-interactome, we used an antibody that recognizes both isoforms (α/β) of synuclein protein. We identified 224 potential interaction partners of synuclein protein, (i) stress-dependent partners (51 proteins), (ii) stress-independent interactors (28 proteins), and (iii) stress-sensitive interactors (145 proteins) (Additional file 1: Fig. S3 B & Additional file 2). The identified proteins include known interacting partners of synuclein (e.g. ARP2, 6PGD, G3BP1 among others). Importantly, a majority of the other identified proteins represent previously uncharacterized interacting candidates (Additional file 2).

**Fig. 4:**
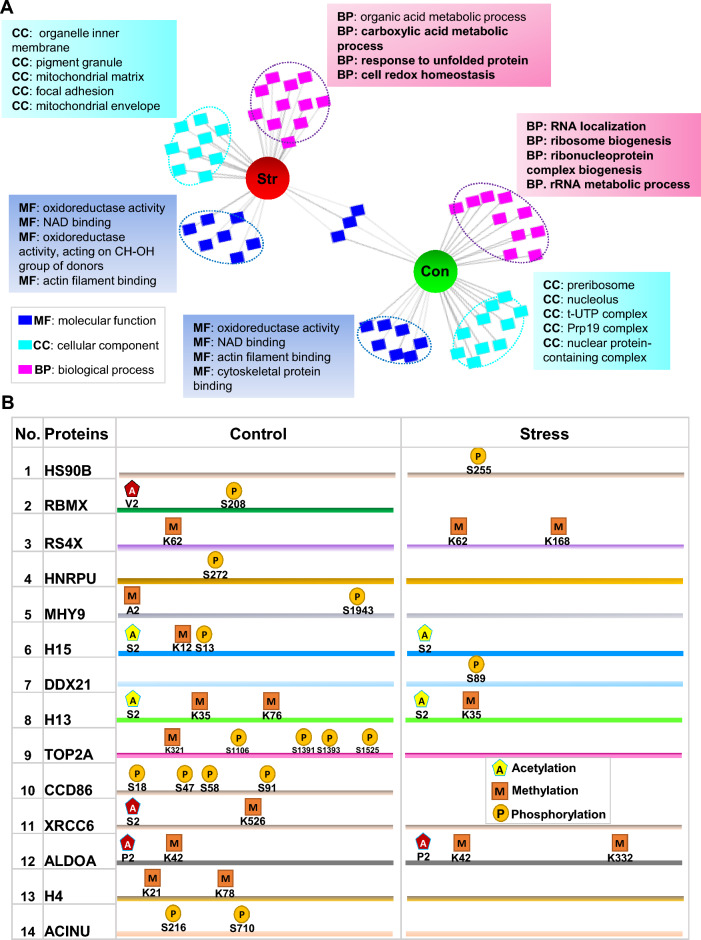
Oxidative stress-induced changes in synuclein interactome. **A** Comparative functional profile of control (con.) and stress (str.) induced cells, in the domains of biological process (BP), molecular function (MF) and cellular component (CC) using ToppCluster. An ‘abstracted’ network option was chosen to generate a Cytoscape-compatible network, containing the top ten enriched terms. Some of the specific terms are labeled in the figure. **B** A map of three posttranslational modifications (Acetylation, methylation, and phosphorylation) identified by mass spectrometry analysis, in both control and stress-dependent interactome of synuclein protein

Intriguingly, stress treatment induced significant alterations in the interactome of synuclein protein. The particular enrichment of ‘‘cell redox homeostasis’’ in the interactome of synuclein protein under stressful conditions indicates the role of synuclein in oxidative stress response (Fig. 4A). Additionally, enrichment of metabolic and actin cytoskeleton-related proteins in the stress-dependent interactome of synuclein protein indicates remodeling of metabolic and cytoskeleton activities as a result of stress stimuli. The significantly enriched categories under control conditions were ‘‘ribosome biogenesis’’, ‘‘protein import into the nucleus’’ and ‘‘ncRNA processing’’ (Fig. 4A). Strikingly, 14 interacting partners of synuclein protein showed stress-mediated changes in PTMs including Heat shock protein HSP 90-beta (HSP90B: phosphorylation-S255), RNA-binding motif protein X chromosome (RBMX: acetylation-V2, phosphorylation-S208), Small ribosomal subunit protein eS4, X isoform (RS4X: methylation-K62 & K168) among others (Fig. 4B).

### Oxidative stress induces changes in the interactome of PrP related to protein localization and DNA-metabolic processes

In total, we identified 230 potential interaction partners of the prion protein, (i) stress-dependent partners (85 proteins), (ii) stress-independent interactors (31 proteins), and (iii) stress-sensitive interactors (114 proteins) (Additional file 1: Fig. S3 C & Additional file 2).

**Fig. 5:**
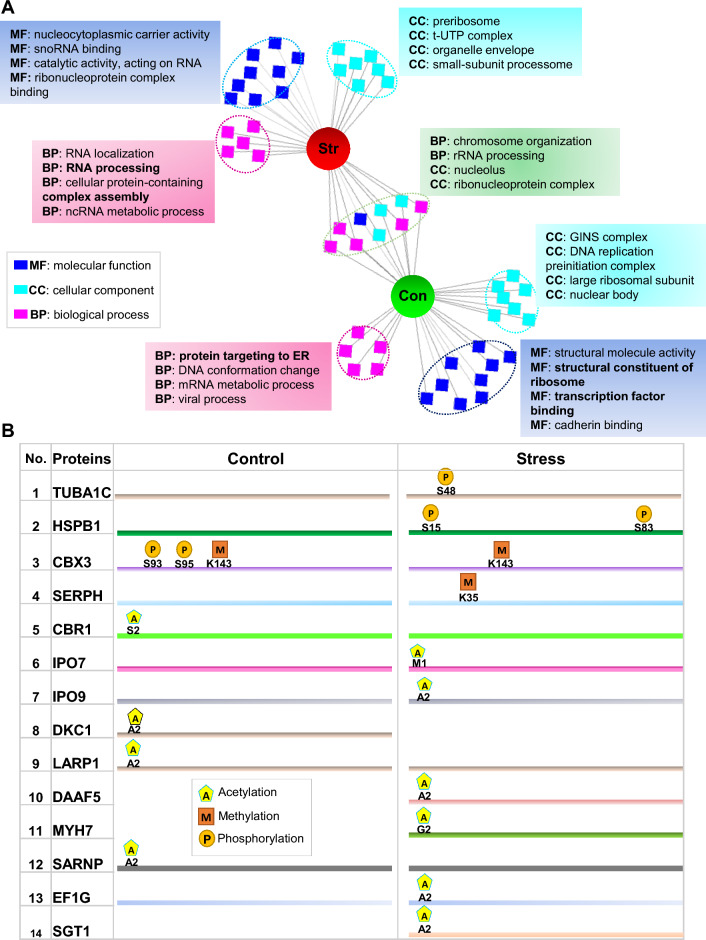
Oxidative stress-induced changes in the prion protein interactome. **A** Functional enrichment analysis for GO-molecular function (MF), -biological process (BP), and -cellular component (CC) in unstressed (con: control) and oxidative stress (str) induced HeLa cells. An abstracted network is showing enriched functional categories in the interactome of PrP. Cytoscape software was used as a visualization tool for the network. The middle circle is showing shared terms. **B** A map of post-translation modifications (phosphorylation, methylation, and acetylation) identified in stress-independent (control) or stress-dependent (stress) interactome of PrP

Functional enrichment analysis of interacting partners of prion protein showed two major themes related to the ‘’DNA-metabolic process’’ and ‘’protein localization’’ that were enriched in control cells. Stress treatment induced significant changes in the interactors of the prion protein. Top-enriched functional categories in the stress-induced cells were related to ‘’RNA localization’’ and ‘’ncRNA processing’’ (Fig. 5A). A map of post-translational modifications that were exclusively identified in either unstressed (control) or stress-induced (stress) cells including Tubulin alpha-1C chain (TUBA1C: phosphorylation-S48), Heat shock protein beta-1 (HSPB1: phosphorylation-S15, S83) and Chromobox protein homolog 3 (CBX3: phospho-S93, S95, methylation-K143) among others. A detailed description is given in Fig. 5B.

### Validation of PrP interaction with exportin-5 in SH-SY5Y Cells

To independently validate the PrP–exportin-5(XPO-5) interaction observed from PrP IP-MS (Additional file 2), we used PrP and XPO5 as bait proteins in immunoprecipitation and reverse co-immunoprecipitation (CO-IP). Exportin-5 was an interesting candidate as cellular stress has been linked with nucleocytoplasmic transport defects due to sequestration of transport components into stress granules [42]. To explore this, we used Co-IP and Co-immunofluorescence under control and stress induced conditions.

**Fig. 6:**
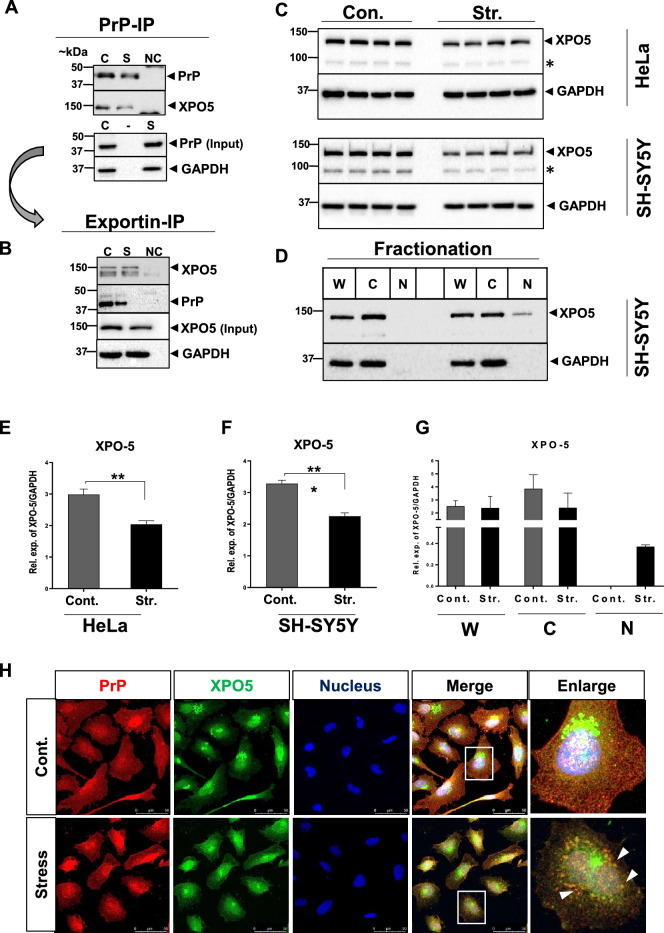
Validation of PrP and XPO5 interaction. **A** Endogenous PrP (bait) was immunoprecipitated from a human neuroblastoma cell line (SH-SY5Y) and exportin-5 (prey) was probed by western blotting in unstressed cells (control: C), stressed cells (stress: S) and negative control (NC) IgG precipitates. **B** Reverse CO-IP: endogenous XPO5 (bait) was immunoprecipitated and probed with PrP (prey) by western blotting. **C** Abundance of XPO5 in HeLa and SH-SY5Y cells under control (cont.) and stress (str.) condition by immunoblotting. *We are not sure about the identity of these bands. **D** Fractionation in SH-SY5Y cells under control and stress-induced condition. W: whole cell fraction, C: cytoplasmic fraction, N: nuclear fraction. **E** and **F** Densitometry analysis of XPO5 in HeLa cells (Welch’s t-test p-value = 0.0052) and SH-SY5Y cells (Welch’s t-test p-value = 0.0004). **G** Densitometry analysis of all fractions in control and stress conditions. **H** Localization of endogenous PrP and XPO5 in stress and control (cont.) HeLa cells using confocal microscopy. Counter-staining was performed to visualize cell nuclei, scale bar = 50 μm

Endogenous PrP (bait) was immunoprecipitated from a human neuroblastoma cell line (SH-SY5Y) and XPO5 (prey) was probed by western blotting. Exportin-5 robustly interacted with PrP in both unstressed (control) and stressed cells (stress) (Fig. 6A) but did not interact with the negative control (NC) IgG precipitate (Fig. 6A). We employed reversed CO-IP to confirm this interaction. Endogenous exportin-5 (bait) was immunoprecipitated and probed with PrP (prey) by western blotting (Fig. 6B).

There were slight differences in the co-purified proteins under control and stress conditions. This prompted us to investigate their expression under control and oxidative stress conditions (Additional file 1: Fig. S2). The abundance of PrP (SAF70) was significantly reduced after stress induction (Additional file 1: Fig. S2B & D) in SH-SY5Y cells. There was also a significant reduction in the mono-glycosylated form of PrP (SAF 32) after stress induction (Additional file 1: Fig. S2B and F). The abundance of exportin-5 was significantly reduced after stress treatment in both HeLa (Fig. 6C and E) and SH-SY5Y (Fig. 6C and F) cell lines. To confirm if decreased intensity levels of exportin-5 protein under stressful conditions were really a decrease in its concentration or simply a decrease in its intensity due to consolidation in SGs after stress treatment, we carried out subcellular fractionation. Remarkably, under control conditions, exportin-5 was only detected in whole cell and cytoplasmic fractions but not in nuclear fractions by immunoblotting. Strikingly, after stress treatment, exportin-5 was detected in nuclear fraction as well (Fig. 6D and G).

Overall, these findings indicate stress-mediated redistribution of XPO5 and its sequestration into PrP-positive foci.

We then examined the colocalization between PrP–XPO5 by performing immunofluorescence using antibodies specific to each protein. In control cells, XPO5 was localized mainly in the perinuclear region in HeLa cells, whereas PrP (SAF32) protein was detected in both, cytoplasmic and nuclear regions. Following stress treatment, XPO5 translocated into distinct granules localized around the nucleus (Fig. 6H). The cytoplasmic exportin-5 and PrP proteins colocalized in the granules after stress treatment (yellow dots in the enlarged image), although some of the XPO5, especially nuclear XPO5, remained in distinct regions, as shown by the green signal (Fig. 6H). Thus, these yellow dots show the partial co-localization of exportin-5 and PrP in the granules.

## Discussion

In the current study, we set out to compare the interactomes of prion/prion-like proteins and associated biological pathways modulated as a result of oxidative stress. To the best of our knowledge, the current study represents the most comprehensive network rearrangements in the endogenous interactome of PrP, tau, and synuclein protein, in response to oxidative stress stimuli. Oxidative stress is something that happens with brain aging, and the inability to deal with it properly contributes to neurodegenerative disorders including AD and PD [43, 44].

Firstly, in the current study, we discovered the localization of PrP and synuclein protein in TIA1-positive stress granules after oxidative stress induction. Considering the involvement of the stress granule pathway in the aggregation of TDP43, FUS, and tau protein, the presence of PrP and synuclein proteins in SGs could have potential pathological consequences. Stress granule formation is mediated via a process called liquid–liquid phase separation. Indeed, full-length PrP can undergo kosmotropic anion, Amyloid-β oligomer, or nucleic acid-induced phase separation [45–47]. Recent evidence indicates that LLPS of α-synuclein precedes its aggregation, in cellular models [48]. In the current study, the localization of synuclein in SGs further strengthens the involvement of SG biology in the aggregation of synuclein protein. Short-term arsenic exposure induced inclusions of synuclein protein [49]. However, the nature of these inclusions was unknown. In the current study, the localization of synuclein inclusions within TIA1-positive SGs suggests that these might be stress granules.

Identificaton of several known interactors (e.g. MAP4, G3BP1, HMGB2, ANXA2, DLDH; Additional file 2) in interactomics data relevant to neurodegenerative disorders indicates that our data is of high quality and important for pathogenesis. Of the 155 tau-interactors, 51 interactors have been previously characterized to interact with tau (32%), further validating our results.

The enrichment of ‘‘Ribonucleoprotein complex biogenesis and rRNA processing’’ in the PrP and tau interactomes after stress-induction, indicate a role of tau and PrP in the genesis of RNP-complexes/granules. Indeed, recently a clear linkage of stress granules with tau pathophysiology has been discovered [23]. The similar network rearrangements in both proteins after oxidative stress, suggest that common adaptive pathways are initially activated by protein interactions, and may progress to pathological responses such as cell death depending on the levels of ROS or oxidative stress induced by the continuous disease stress. A protective role of prion protein against oxidative stress has also been documented [50, 51]. A shift from DNA metabolism-related processes to RNA catabolic processes after oxidative stress, including ncRNA processing (25 proteins) may have important implications for PrP pathological conversion [52].

Remarkably, biological processes ‘‘RNP complex and ribosome biogenesis’’ were lost after stress induction in synuclein interactome, indicating an impairment of protein synthesis machinery after stress exposure. Indeed a region-dependent impairment in protein synthesis machinery has been reported in PD [53]. An enrichment of ‘‘cell redox homeostasis’’ suggest a linkage of synuclein and its interactome with redox homeostasis[54]. An important finding was the enrichment of metabolism-related processes showing a linkage of synuclein with metabolic interface during stressful conditions.

Post-translational modifications of proteins are a rapid and efficient way to quickly yield proteins with new features demanding relatively little cellular energy [55]. In the current study, acetylation was the most frequent modification observed in the MS-detected modifications. Around 85% of human proteins are believed to be N-terminally acetylated[56]. Our results show methylation of XRCC5 protein (interacting candidate of tau proteome) at Lysine (K702). To the best of four knowledge, this is the first report showing MS-based evidence for this novel PTM of XRCC5, which was lost after stress treatment. Acetylation of Importin-7 and Importin-9, under stressful conditions, suggest modulation of nuclear import via PTMs under stressful conditions.

An interaction of PrP with exportin-5 by CO-IP and immunocytochemistry suggest a possible way of nucleo-cytoplasmic transport of PrP protein or its RNA. Although, co-immunoprecipitation and co-localization experiments will not definitively determine whether these protein–protein interactions are direct. There is a possibility that additional interacting partners can mediate the identified interactions [57]. Dysregulation of PrP mono-glycosylated form after sodium aresnite-induced stress, suggests specific involvement of post-translationally modified forms of PrP in stress response. Strikingly, downregulation and nuclear accumulation of XPO5, as a result of stress stimuli suggests nucleo-cytoplasmic transport defects during the stress response. Interestingly, an additional band between 100- and 75-kDa (denoted by star) was recognized by the exportin 5 antibody as an untargeted protein due to the cross reaction. This finding suggest that fragmented exportin 5 may also occur in these cells, however future investigations are required to confirm the identity of these bands.

In this study, stress was induced in HeLa cell model. Several cellular models have been used to understand pathophysiology of SGs at cellular level [10]. A significant amount of knowledge on stress granules is generated from investigations in HeLa cells, including 154 studies published between 1999 and 2014 [10]. Although, this is a non neuronal cell line, this model worked for this exploratory study as bigger cytoplasmic area of HeLa cells in comparison with other cell lines allows accurate identification of cytoplasmic SGs [18]. Furthermore, validations of interactomics data in the neuroblastoma cell line (SH-SY5Y) indicates reproducibility and reliability of our interactomics data.

The current study has established a link between stress granules and prion/prion-like proteins, especially PrP and synuclein proteins, and nuclear accumulation of XPO5. Future investigations are required to find out what drives PrP and synuclein translocation into stress granules, and pathophysiological significance of these granules. We believe that further investigations of these granules under chronic stress conditions may shed light on misfolding events of these proteins, as persistent stress granules have been linked with the misfolding and aggregation of tau protein [5].

## Conclusions

Overall, using interactomics and network rearrangements, the current study broadens our understanding of the pathophysiological context of aggregated-prone proteins. The current study provides a full map of interactome readjustments of aggregation-prone proteins during oxidative stress condition. Localization of prion protein and synuclein in the cytoplasmic SGs provides an important link between stress granule pathobiology and aggregation-prone proteins. In addition, our findings demonstrate nucleocytoplasmic transport defects after oxidative stress via dysregulation and nuclear accumulation of exportin-5. Further investigation of the linkage of prion protein and synuclein with stress granule pathobiology might highlight novel pathways involved in the aggregation of these proteins. These investigations could lead to the identification of a core pathological process involved in disease-related aggregation. In addition, the current study provides a new category for exploration ꟷstress-induced PTMsꟷ which are very important to understand the complex pathobiology of these aggregation-prone proteins. Findings from the current study provides an exciting roadmap to find out new biomarkers and therapeutic targets for neurodegenerative diseases.

## Methods

### Cell culture and stress induction

Neuroblastoma cell line, SH-SY5Y [23, 58], and HeLa cell line (CCL-2—ATCC) were cultured and maintained in Dulbecco’s modified DMEM supplemented with fetal bovine serum (10%) and penicillin–streptomycin (1%) at 37 °C with 5%. For SH-SY5Y cells, media was supplemented with GlutaMax (Gibco™). To induce stress, cells were exposed to media supplemented with 0.6 mM of sodium aresnite for one hour at 37 °C.

### Cell lysis and Co-immunoprecipitation

Cell lysis was performed as described previously [23]. Briefly, cells were lysed (50 mM Tris–HCl, pH 8, 1% Triton X-100, 0.5% CHAPS, 1 mM DTT) on ice with protease and phosphatase inhibitors (Roch) followed by incubation at 4 °C for one hour with shaking to completely lyse them. To get rid of the cell debris, the lysates were centrifuged at 14000 rpm for 30 min at 4 °C. The co-immunoprecipitation was performed using magnetic Dynabeads protein G (Invitrogen), according to the manufacturer’s protocols. Altogether, we performed 30 co-immunoprecipitations of endogenous target proteins (PrP, synuclein and tau) with corresponding negative controls in HeLa cells under control and oxidative stress conditions followed by mass spectrometry analysis (Additional file 1: Fig. S1 A). All the bait proteins were tested in 10 biological replicates (4 biological replicates from control and 4 from stress conditions with corresponding negative controls. For mass spectrometry analysis two technical replicates were run for each biological replicate (total technical replicates, n = 60). All the validations were performed in neuroblastoma cell line (SH-SY5Y). Co-immunoprecipitations and reverse CO-IPs for validation studies were carried out in SH-SY5Y cells.

### Immunoblotting analysis

Immunoblotting analysis was carried out as reported previously [23]. Briefly, total cell lysates or co-immunoprecipitates were electrophoresed onto a 12% one-dimensional SDS-PAGE (in-house prepared gels) or 4–12% Bis–Tris gels (NuPAGE™ 4–12% Bis–Tris Protein Gels, Invitrogen). The expression of target proteins was analyzed by immunoblotting using overnight exposure at 4 °C to primary antibodies including anti-PrP monoclonal antibody (SAF32, 1:1000, #A03202), anti-PrP mAb (SAF70, 1:1000, #A03206), anti-α/β synuclein mAb (1:1000, #2644S), anti-exportin-5 mAb (1:1000, #12565), and anti-GAPDH polyclonal antibody (1:1000). Images were acquired using Chemi-Doc (Bio-Rad) by developing membranes into enhanced chemiluminescence solution. The band intensities were determined by densitometric analysis using Image Lab software (version 3.0.1).

### Label-free mass spectrometry (LC–MS/MS) analysis

Mass spectrometry analysis was carried out as published previously [23]. Briefly, co-immunoprecipitates were run onto 4–20% Bis–Tris gels (Invitrogen) for a length of ~ 1 cm. After Coomassie Brilliant Blue staining, gel bands were excised from the gel and in-gel digested into peptides. These peptides were identified as described previously. Scaffold software (version Scaffold_4.11.0) was used to validate MS/MS-based peptide and protein identifications. Protein identifications were established at an FDR less than 1% and with at least 2 identified peptides. To assign protein probabilities the Protein Prophet algorithm was used [59].

### Bioinformatics analysis

To gain functional insights from the identified interactome, functional enrichment analysis was performed using different overrepresentation tools. Functional network mapping was performed using ToppCluster and ShinyGO. ToppCluster is a web-based tool for comparative analysis of multiple gene lists simultaneously for functional enrichment analysis on large-scale data sets [60]. ToppCluster yields output as a rich functional map showing the shared and unique functional features among different input gene lists. Most of the available functional enrichment tools analyse a single gene list at a time, while ToppClusters offers the possibility of co-analysis of multiple gene lists, which is very useful to get a comparative view of shared and unique functions under different conditions. A Cytoscape-compatible network was generated, containing the top ten enriched term relationships. The functional maps were edited using Inkscape (version 0.92). For functional enrichment analysis, total interacting partners (Additional file 1: Figure S3) under control and stress conditions were used from each bait protein (Additional file 1: Fig. S3 & Additional file 2).

### Granule formation analysis via catGRANULE algorithm

To analyse the granule formation tendency of PrP and synuclein protein, we used catGRANULE algorithm, which calculates the tendency of a protein to undergo phase separation and the probability to form granules [25]. The algorithm measures the granule formation propensity of a protein based on its nucleic acid binding properties, structural disorder and, to a lesser extent, amino acid pattern (length and content of arginine, glycine, and phenylalanine) which are particularly enriched in granule forming proteins [61, 62]. A score > 0 indicates that a protein is prone to phase separate and a score > 1 shows high-confidence phase separation tendency of a protein [25].

### Immunofluorescence, imaging, and image analysis

Immunofluorescence was performed as described previously [23]. Briefly, after stress induction, cells were fixed in 4% formaldehyde for 20 min, followed by permeabilization for 10 min with 0.5% Triton-X. After 3 × washes with PBS, cells were blocked with blocking buffer (1 × PBS, 1%BSA, 10% FBS). Cells were incubated with the primary antibodies against stress granule markers like TIA1 (sc-1751), exportin-5 (12565), PrP antibodies (SAF32: #A03202, SAF 70 #A03206: 12F10: #A03221), and α/ꞵ synuclein (sc-69699) overnight at 4 °C. The cells were thoroughly washed with PBS, and incubated for 2 h with secondary antibodies (Alexa Fluor 488, # A-11001, Alexa Fluor 488, # A-11008, Alexa Fluor 555, # A-21424, Alexa Fluor 546, # A-11010, Invitrogen) diluted in blocking buffer (1%BSA/PBS). RedDot2 far red (Biotium) was used for nuclear staining. Imaging was performed using a confocal laser-scanning microscope (TCS-SPE, Leica).

### Subcellular fractionation

Cellular fractionation was performed as described previously [63]. Briefly, confluent SH-SY5Y cells were washed with cold PBS, scraped, and resuspended in 1 mL PBS. This cell suspension was separated into whole-cell, nuclear and cytoplasmic fractions. From each fraction, 10 μL were used for immunoblotting analysis with primary antibodies specific for exportin-5 and GAPDH.

### Statistical analysis

All the data in this study was obtained from at least three independent experiments. Statistical analysis was performed using GraphPad Prism 6.01. All the results are reported as mean ± SEM (standard error of the mean). The data from mass spectrometry were processed and analyzed using the Perseus software. Statistical significance was determined for a p-value < 0.05.

### Supplementary Information

**Additional file1: Figure S1.** Co-immunoprecipitation (CO-IP) and mass spectrometry analysis of tau, PrP, and synuclein interactome. **A** Workflow of Immunoprecipitation mass spectrometry (IP-MS) for high throughput screening of interactome. The abundance of proteins co-purifying with the selected target bait proteins was compared between control and oxidative stress-induced (sodium arsenite treatment) cells followed by functional analysis using different bioinformatic tools. **B** Immunoprecipitation of native, endogenous bait proteins using anti-SAF70, anti-tau-5, α/β-synuclein antibodies. Neg. con: Negative control IGg. FL: Full-length antibody, HC: Heavy chain of the antibody, LC: light chain of the antibody. **C **A Comparison of all three bait protein interactomes shows similar and unique proteins in all groups. The candidate interactors from all three bait-proteins were uploaded to Venny Web tool (2.1.0) for intergroup comparisons under control and stress conditions. The horizontal bars are showing unique interactors (in percentage format) from each bait protein. The vertical bars are showing shared interactors (left boxes: proteins shared among all three bait proteins, right-side boxes: proteins shared between two bait proteins). **Figure S2. **Analysis of expression of prion protein and synuclein after oxidative stress treatment (sodium arsenite).** A**, **B **HeLa and SH-SY5Y cell lines were treated with sodium arsenite (0.6 mM, 60 min), Expression of prion protein (anti-SAF 70 and anti-SAF32) and α/β-synuclein was analyzed by immunoblotting in both control (con.) and sodium arsenite treated cells (str.). GAPDH was used for normalization. **C**–**F** Quantification of all the proteins is showing significantly decreased intensity levels for PrP-SAF70 (Welsch’s t-test p value = 0.0309) and mono-glycosylated form of PrP (SAF32, Welsch’s t-test p value = 0.0090). **Figure S3. **The candidate interacting partners identified under control and stress conditions for each bait protein.** A**–**C **Venn diagrams showing interacting partners (shared and unique) between control and oxidative stress-induced (sodium arsenite treatment) cells for three bait proteins (tau, PrP and synuclein proteins).

**Additional file 2.** Interacting proteins identified by mass spectrometry analysis in all three bait proteins (tau, PrP, and synuclein) under basal and stress-induced conditions (sodium arsenite treatment). Stress-dependent partners: Proteins, which associate with the bait protein only upon oxidative stress induction. Stress-independent interactors: Proteins, which associate with the bait protein independently of stress. Stress-sensitive interactors: which are lost after stress induction.

## Data Availability

All data generated or analyzed during this study are included in the main article and its Additional files.

## Abbreviations

AD: Alzheimer’s disease
ALS: Amyotrophic Lateral Sclerosis
CO-IP: Co-immunoprecipitation
FDR: False discovery rate
LLPS: Liquid-liquid phase separation
MS: Mass spectrometry
SGs: Stress granules
PD: Parkinson’s disease
PrP: Prion protein
PTMs: Posttranslational modifications
XPO5: Exportin-5
