# Supplementary figures and images for: Differential interactome mapping of aggregation prone/prion-like proteins under stress: novel links to stress granule biology
Source: Cell Biosci. 2023 Dec 1;13:221. doi: 10.1186/s13578-023-01164-7 (PMC10693047; doi:10.1186/s13578-023-01164-7)

**A**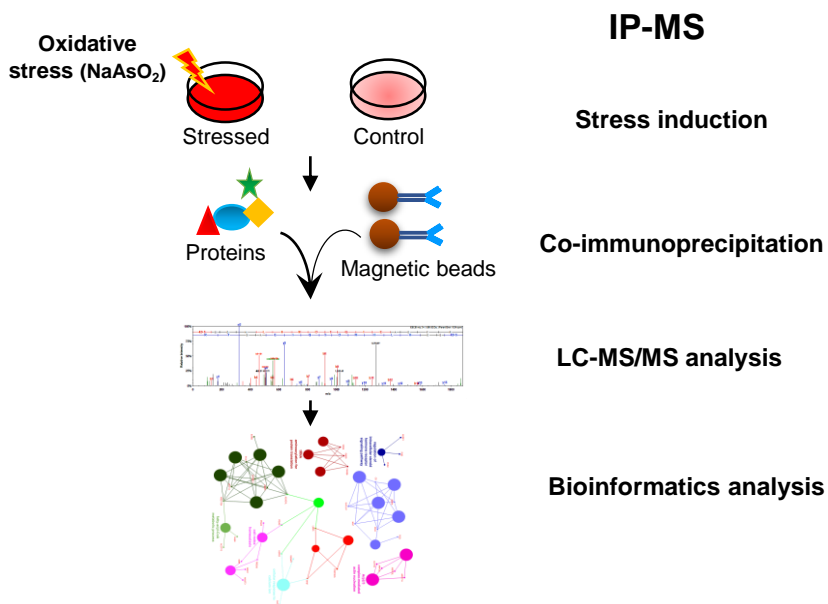**B**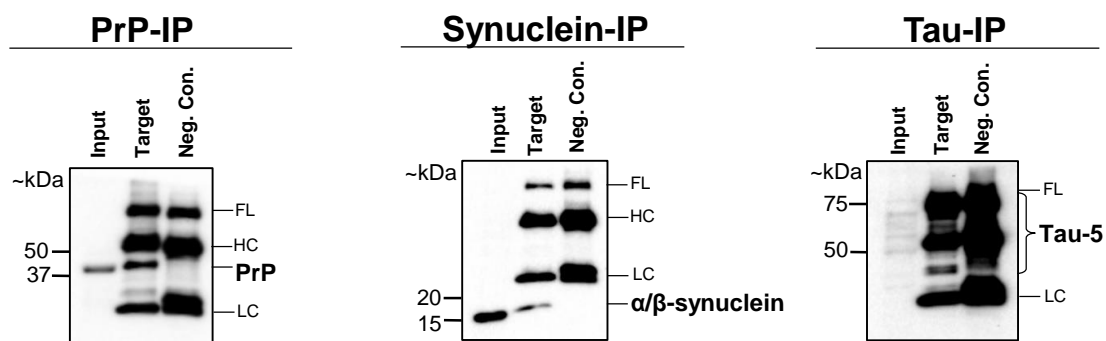**C**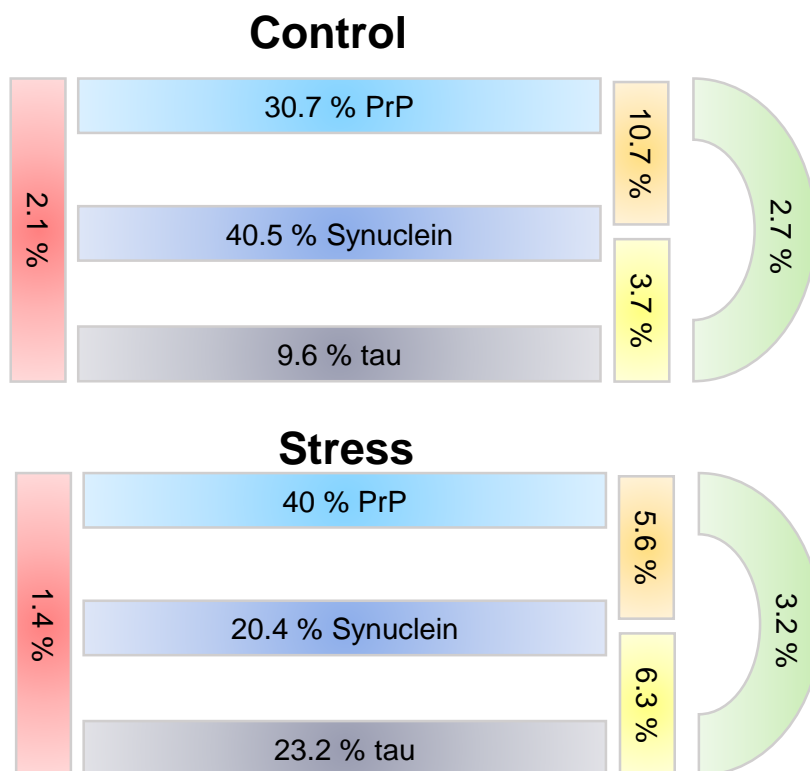**Fig. S1**

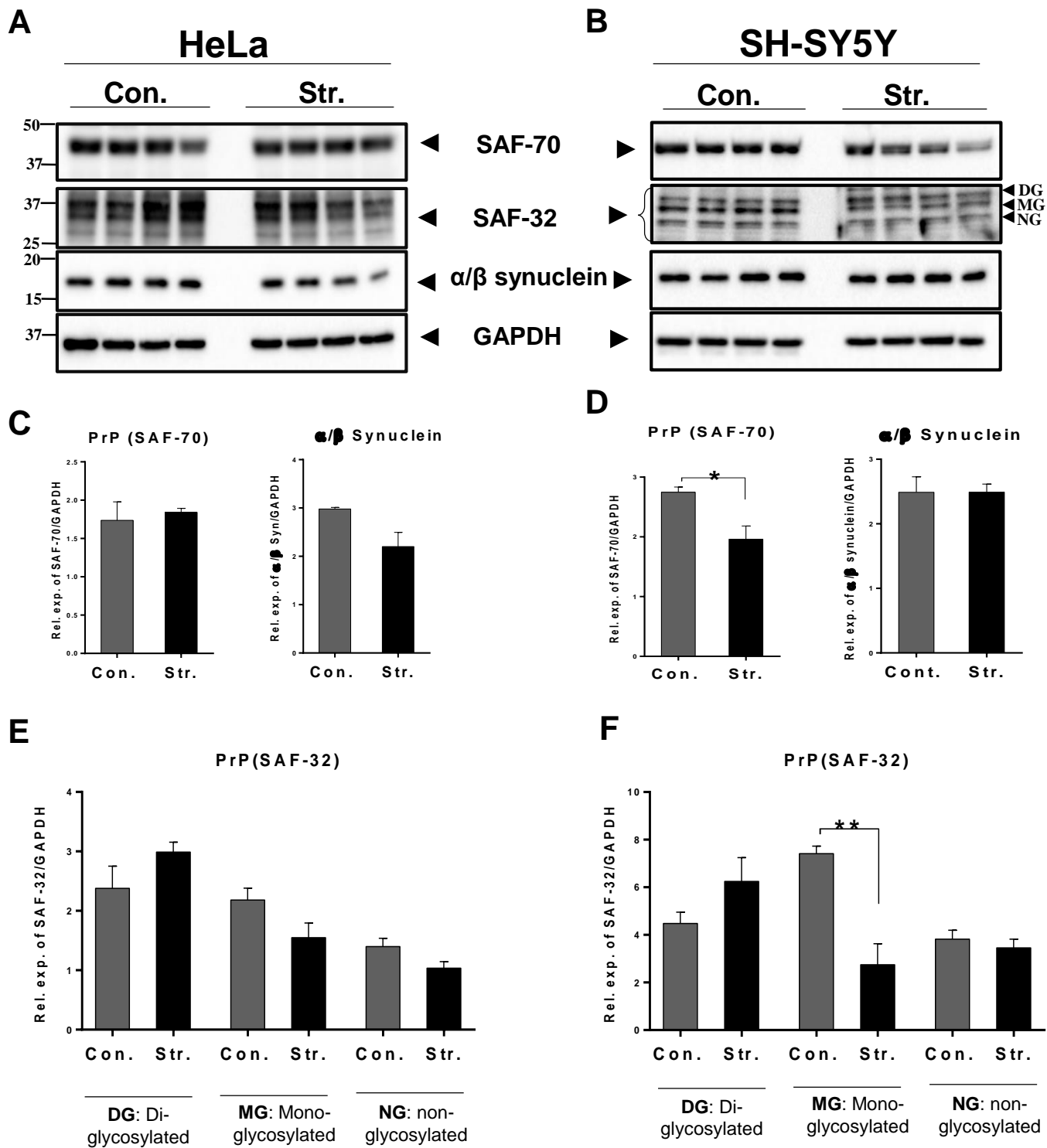

**Fig. S2**

## **Tau (143)**

**A**

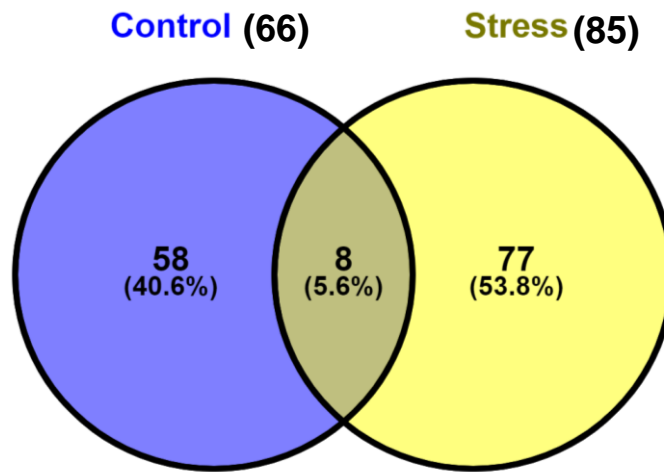

**B**

## **PrP (230)**

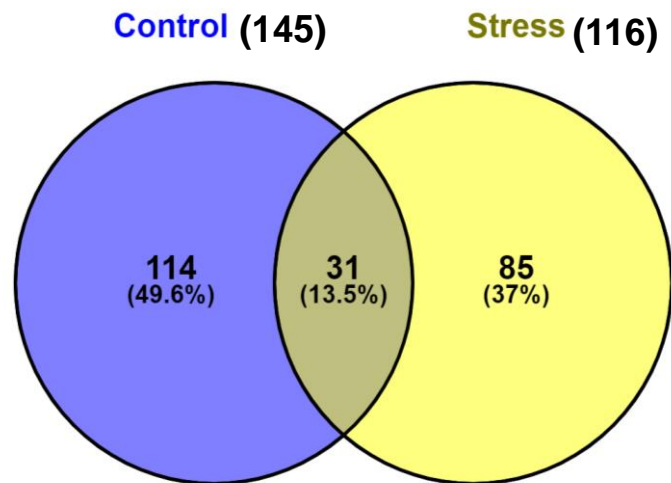

**C**

## **Synuclein (224)**

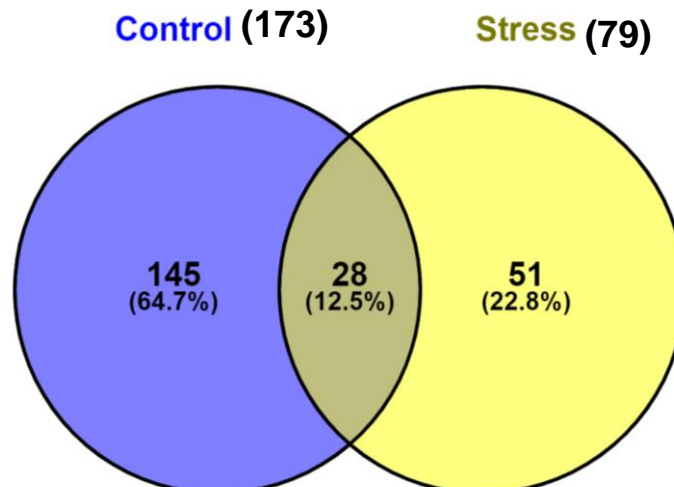

**Fig. S3**

Supplement: Supplementary file 1 — Additional file1: Figure S1. Co-immunoprecipitation (CO-IP) and mass spectrometry analysis of tau, PrP, and synuclein interactome. A Workflow of Immunoprecipitation mass spectrometry (IP-MS) for high throughput screening of interactome. The abundance of proteins co-purifying with the selected target bait proteins was compared between control and oxidative stress-induced (sodium arsenite treatment) cells followed by functional analysis using different bioinformatic tools. B Immunoprecipitation of native, endogenous bait proteins using anti-SAF70, anti-tau-5, α/β-synuclein antibodies. Neg. con: Negative control IGg. FL: Full-length antibody, HC: Heavy chain of the antibody, LC: light chain of the antibody. C A Comparison of all three bait protein interactomes shows similar and unique proteins in all groups. The candidate interactors from all three bait-proteins were uploaded to Venny Web tool (2.1.0) for intergroup comparisons under control and stress conditions. The horizontal bars are showing unique interactors (in percentage format) from each bait protein. The vertical bars are showing shared interactors (left boxes: proteins shared among all three bait proteins, right-side boxes: proteins shared between two bait proteins). Figure S2. Analysis of expression of prion protein and synuclein after oxidative stress treatment (sodium arsenite). A, B HeLa and SH-SY5Y cell lines were treated with sodium arsenite (0.6 mM, 60 min), Expression of prion protein (anti-SAF 70 and anti-SAF32) and α/β-synuclein was analyzed by immunoblotting in both control (con.) and sodium arsenite treated cells (str.). GAPDH was used for normalization. C–F Quantification of all the proteins is showing significantly decreased intensity levels for PrP-SAF70 (Welsch’s t-test p value = 0.0309) and mono-glycosylated form of PrP (SAF32, Welsch’s t-test p value = 0.0090). Figure S3. The candidate interacting partners identified under control and stress conditions for each bait protein. A–C Venn diagra [file 13578_2023_1164_MOESM1_ESM.pdf]
